# Determinants of emotional distress in neonatal healthcare professionals: An exploratory analysis

**DOI:** 10.3389/fpubh.2022.968789

**Published:** 2022-09-29

**Authors:** Sarah Nazzari, Serena Grumi, Sabina Ciotti, Ilaria Merusi, Livio Provenzi, Luigi Gagliardi

**Affiliations:** ^1^Department of Brain and Behavioral Sciences, University of Pavia, Pavia, Italy; ^2^Developmental Psychobiology Lab, IRCCS Mondino Foundation, Pavia, Italy; ^3^Division of Neonatology and Pediatrics, Ospedale Versilia, Viareggio, Italy; ^4^AUSL Toscana Nord Ovest, Pisa, Italy

**Keywords:** neonatal healthcare professionals, stress, coping, safety culture, NICUs, behavioral activation, behavioral inhibition

## Abstract

**Background:**

High levels of mental health problems have been consistently reported among neonatal healthcare professionals. While studies suggest that personality, coping strategies and safety culture might contribute to the psychological wellbeing of healthcare professionals, they have not been systematically investigated in low-risk (i.e., neonatal wards; NWs) and high-risk (i.e., neonatal intensive care units; NICUs) neonatal contexts. The current study investigated potential predictors of professionals' emotional distress and whether they differ according to the work setting (i.e., NICUs vs. NWs).

**Methods:**

Healthcare professionals (*N* = 314) from 7 level-3 (i.e., NICUs) and 6 level-2 (i.e., NWs) neonatal units in Tuscany were included. Emotional distress (i.e., anxiety, depression, psychosomatic, post-traumatic stress symptoms and emotional exhaustion), Behavioral Inhibition System (BIS) and Behavioral Approach System (BAS) sensitivity, coping strategies and safety culture were assessed through well-validated, self-reported questionnaires.

**Results:**

Greater BIS/BAS sensitivity, avoidance coping strategies and a sub-dimension of safety culture (i.e., stress recognition) were significantly associated with greater risk of emotional distress, whereas job satisfaction emerged as a protective factor. Three specific profiles of professionals in term of personality, coping and safety culture were identified and further predicted emotional distress. Neonatal wards and NICUs personnel presented different associations between personality, coping and safety culture.

**Conclusion:**

These findings highlighted significant modifiable contributors of neonatal mental healthcare professionals' wellbeing. Institutional initiatives that target these factors and, particularly, job satisfaction may promote professionals' emotional wellbeing and thus improve caring processes.

## Introduction

The healthcare environment is inherently complex, demanding, and stressful. Stress experienced by healthcare professionals, especially by physicians and nurses, results from their responsibility for health and wellbeing of other people, patients' behavior and complaints, coping with death and injury (1, 2). Among healthcare professionals, those who work in neonatal contexts and, particularly, in neonatal intensive care units (NICUs), are exposed to an additional amount of stress related to caring for the youngest and for their parents in very at-risk situations and to exposure to potentially traumatic events (3). Furthermore, the ever-mounting business competition, together with the recent health emergency, have led to an enormous increase in the amount of workload, job pressure and stress on professionals working in these settings (4–6). Thus, serious concerns have been raised for the psychological wellbeing of this occupational group which might have a cascade impact on the quality of services provided (7).

Estimates showed that a significant proportion of neonatal and pediatric healthcare professionals reports high levels of anxiety, depression, psychosomatic symptoms, post-traumatic stress symptoms, and emotional exhaustion (8–10). Professionals working in the NICUs have been reported to be at higher risk of developing emotional distress as compared to their colleagues working in lower-risk settings such as neonatal wards (NWs) (11, 12). Emotional distress is defined in literature as a “multi-factor, general mood disorder defined as subjective feelings that vary in intensity from sadness, uncertainty, confusion and worry to more significant symptoms such as anxiety, the expression of anger, social isolation and hopelessness” (13). The recent sanitary crisis due to the COVID-19 pandemic has further raised the rate of mental health problems among healthcare workers reaching epidemic levels (14), even in neonatal care settings (15–17).

Notably, not all individuals develop mental health problems in response to stress conditions. The way individuals experience, approach, appraise, and manage stress can make a difference. This means that personality characteristics, coping strategies and perception of working safety have the potential to provide an explanation as to why some individuals thrive under stress conditions, while for other the same stressful experience may disrupt their physical and mental health wellbeing. The current study investigated how personality, coping and safety culture associate with emotional distress in healthcare professionals from NWs and NICUs settings.

The Reinforcement Sensitivity Theory [RST, (18, 19)] is a biologically-based theory of personality that might offer one of the most accurate descriptions of the link between personality and psychopathology (20, 21). According to this theory, individuals differ in their reinforcement sensitivity which reflects the activity of two basic brain sub-systems, the Behavioral Inhibition System (BIS) and the Behavioral Approach System (BAS). The BIS is associated with response to threat by motivating withdrawal behavior, whereas the BAS is associated with reward-seeking behavior by motivating approach behavior. Extreme under- or over-sensitivity of these systems is proposed to predict psychopathology (19), with high BIS sensitivity being considered a risk factor for internalizing symptoms, whereas elevated BAS activity being hypothesized to increase vulnerability to externalizing problems in the general population (20).

Coping strategies refer to the behavioral and cognitive efforts put in place to manage stressful situations (22) and are important factors that might modulate individuals' responses to stress in the workplace. A study reported that the most common coping strategies used by healthcare workers included acceptance of the critical situation and use of a positive outlook while working (23, 24). Importantly, distinct coping strategies have been found to, respectively, increase or reduce the risk of emotional distress in healthcare workers (25, 26). Specifically, a positive attitude, problem solving and seeking social support are regarded as adaptive coping styles (27) and they have been associated with reduced emotional distress (28), stress symptoms (29), anxiety and depression (30). In contrast, avoidance strategies are considered negative coping styles, being associated with an increase of psychological distress (31), emotional exhaustion (32), post-traumatic stress symptoms and fatigue (33).

Safety culture, as the professionals' attitudes and behavior about the organization's current health and safety performance (34), is strongly related with healthcare workers' wellbeing (35). Mounting evidence indicates that healthcare environments in which professionals have autonomy, control over the environment and good relationships within the team result in lower levels of burnout (36). This is even more critical in high-risks settings such as the NICUs. Safety culture within the NICUs has been shown to vary widely (37) and one study indicates that it might negatively associate with NICUs nurses' burnout (8).

While studies suggest that personality, coping strategies and safety culture might predict the occurrence of burnout syndrome in health professionals (28, 35, 38), they have not been systematically investigated in relation to professionals emotional distress, encompassing a broader range of mental health outcomes, and within neonatal contexts. A better understanding of factors that contribute to neonatal healthcare professionals' emotional wellbeing will enable to develop prevention strategy and has important implications for the quality of care provided to the youngest. Based on these premises, the aim of the current study was twofold: (1) to examine the associations between personality factors, coping strategies and safety culture and professionals' emotional distress and exploratorily identify specific profiles of professionals (2) to investigate whether the contributors to professionals' emotional distress differ according to the work setting (i.e., NICUs vs. NWs). We hypothesized lower BIS/BAS scores, adaptive coping strategies and a stronger safety culture to be negatively associated with workers' emotional distress. Due to the lack of available evidence, aims 2 was exploratory and we made no a priori predictions.

## Methods

### Participants and procedures

Healthcare professionals (*N* = 314) were recruited as part of the SPACE-NET project (16), a multicentre cross-sectional survey whereby questionnaires were distributed to doctors and nurses across 7 level-3 neonatal units (i.e., NICUs that provide intensive care to newborns <32 weeks of gestation or <1,500 g birth weight, NICUs) in Tuscany, and all 6 level-2 neonatal units (i.e., NWs that provide care to infants ≥32 weeks or >1,500 g) of AUSL Toscana Nord Ovest. The majority were females (*n* = 281, 89.5%) and working as nurses (*n* = 145, 46.2%) or physicians (*n* = 100, 31.8%). One hundred ninety-two participants were working in NWs (61.1%) whereas the remaining (*N* = 122, 38.5%) were working in NICUs. Healthcare professionals were contacted by email. Those who agreed to participate in the study provided an informed consent and anonymously filled in a set of on-line questionnaires to assess their mental health during the COVID-19 emergency, personality factors, coping strategies and safety culture. Filling in the online survey took approximately 30 min. The study has been approved by the Ethics Committee of the participant parties.

### Measures

#### Emotional distress

Several domains of professionals' mental health were investigated. Depressive symptoms were assessed through the 21-items Beck Depression Inventory – II (BDI-II) (39), a widely employed scale that evaluates subclinical and clinical depressive symptomatology. Each item is rated on a 4-point Likert scale; a global sum score is computed with higher scores indicating higher depressive symptoms. Anxiety symptoms were measured using the 20-items State anxiety subscale of the State-Trait Anxiety Inventory – Y form (STAI-Y) (40). Each item is rated on a 4-point Likert scale and sum up in a global score, with higher scores indicating greater levels of current anxiety symptoms. Psychosomatic symptoms were assessed through the 17-items Psychosomatic Symptom Checklist (41) on a 6-point Likert scale. A mean score was obtained with higher scores indicating greater psychosomatic symptoms. Emotional Exhaustion was measured through a 9-items subscale of the Maslach Burnout Inventory (MBI) (42). Each item is rated on a 7-point Likert scale and sum up in a global score with higher ratings indicating greater emotional exhaustion. Lastly, post-traumatic stress symptoms were assessed using the 22-items Impact of Event Scale (IES) (43). Each item is rated on a 5-point Likert scale and a global sum score is obtained with higher scores indicating greater risk for post-traumatic stress symptoms.

As all mental health domains were moderately to strongly correlated (rs range = 0.27–0.71), to limit the number of statistical comparisons, we employed a principal component analysis (PCA) to estimate a global index of emotional distress (EDI) that would explain the largest portion of variance in mental health outcomes (i.e., depressive symptoms, anxious symptoms, psychosomatic symptoms, post-traumatic symptoms, and emotional exhaustion), as done in prior work (16). For this analysis we set the minimum Eigenvalue to 1 and we adopted a non-rotated solution. The principal component with the highest loading and explaining the highest portion of variance was employed as the primary outcome variable in subsequent analyses. The Principal Component Analysis yielded a one-component solution (i.e., emotional distress index, EDI), explaining the 65.3% of total variance and with loadings ranging from 0.67 to 0.90.

#### BIS and BAS sensitivity

Individual differences in BIS and BAS sensitivity were assessed using the widely employed 20-items Behavioral Inhibition/Activation Scale (BIS/BAS) (44). This include seven items that assess BIS sensitivity (e.g., “I worry about making mistakes” or “I feel pretty worried or upset when I think or know somebody is angry at me”) and 13 that assess BAS sensitivity (e.g., “I'm always willing to try something new if I think it will be fun”) on a 4-point Likert scale. Higher scores reflect higher BIS or BAS sensitivity. The BIS scale is unidimensional, while the BAS items can be scored on three different BAS domains: Reward Responsiveness (BAS-R), that focuses on responses to the occurrence or anticipation of reward (e.g., “When good things happen to me, it affects me strongly”), Drive (BAS-D), concerned with the persistent pursuit of desired goals (e.g., “When I want something, I usually go all out to get it”), and Fun Seeking (BAS-F) which reflects a desire for new rewards (e.g., “I'm always willing to try something new if I think it will be fun”).

#### Coping style

Coping strategies were assessed using the Italian validation of the short version of the Coping Orientation to the Problems Experienced (COPE-NVI-25) (26). The COPE-NVI-25 consists of 25 items evaluating how often individuals use a particular coping strategy in stressful situations. Items are scored on a 4-point Likert scale with higher scores indicating a greater use of that strategy. Individual differences in the use of five substantially independent dimensions of coping are assessed: Social Support (e.g., “I try to get advice from someone about what to do”), Avoidance Strategies (e.g., “I admit to myself that I can't deal with it, and quit trying”), Positive Attitude (e.g., “I look for something good in what is happening”), Problem Solving (e.g., “I focus on dealing with this problem, and if necessary let other things slide a little”) and Trascendent Orientation (e.g., “I pray more than usual”). Good psychometric properties have been reported for this version of the questionnaire (26).

#### Safety culture

Professionals' perception of safety in their workplace was investigated through 22 items from the Safety Attitudes Questionnaire Short Form (SAQ) (45). Four of the six domains measured by the SAQ were used in the present study: Teamwork climate, measuring perceived quality of collaboration between personnel (e.g., “Our doctor and nurses work together as a well-coordinated team”), Job satisfaction, assessing positivity about the work experience (e.g., “I like my job”), Safety climate, that focuses on perceptions of a strong and proactive organizational commitment to safety (e.g., “I would feel perfectly safe being treated here”), and Stress recognition, that measures acknowledgment of how performance is influenced by stressors (e.g., “When my workload become excessive, my performance is impaired”). Items are scored on a 5-point Likert scale with higher scores reflecting more positive perceptions.

### Statistical analyses

Variables were first examined for outliers and skewness. Positively skewed distributions were natural log transformed to approximate normal distributions. All variables found to be significantly associated with the EDI were included as covariates in subsequent analyses. Differences in mean levels of personality, coping and safety between professionals from NICUs or NWs were determined by Student's *t* tests. Forward stepwise multivariate regression analyses were performed to identify significant predictors of EDI. At each step, the predictor that had the highest correlation with the outcome variable were entered in the model, if it satisfied the default criterion (i.e., has a *p* < 0.05). The procedure stopped when there were no variables left that satisfied the entry criterion (i.e., when all remaining variables have a *p* > 0.05 if included in the model). Furthermore, we performed a two-step cluster analysis in order to exploratorily identify specific profiles of professionals in terms of personality, coping strategies and safety culture and their relationship with emotional distress. When the groups or clusters had been identified, a univariate ANOVA was performed to determine the existence of significant differences between the groups with respect to the EDI. To determine which means were significantly different, the Scheffé *post-hoc* comparison test was applied.

Lastly, regression models were performed separately for professionals from NWs and NICUs to assess the impact of working setting on these associations.

For all the statistical tests performed, a *p*-value threshold of 0.05 was set to determine statistical significance. Statistical analyses were conducted using IBM SPSS Statistics for Windows, ver. 25.

## Results

### Preliminary analyses

A total of 314 healthcare professionals participated in the study, out of 941 invited (32.9%). Socio-demographic variables (i.e., sex, job, and years of experiences; see Table 1) were not significantly associated with the EDI (all *p* > 0.05), nor there were statistically significant differences in EDI scores depending on work setting (i.e., NICUs vs. NWs). Spearman bivariate correlations between EDI scores and predictors of interest are reported in Table 2 for the whole sample, NWs and NICUs settings. Descriptive statistics and *t*-test results for personality, coping strategies, and safety culture in NWs and NICUs are reported in Table 3. Significant differences were reported only for SAQ job satisfaction, with professionals from the NICUs reporting higher levels of job satisfaction as compared to professionals from the NWs (*p* = 0.04).

**Table 1:** Socio-demographic variables of the study sample.

			**Setting**				**Job**					
	**All** **(*****n*** = **314)**		**NWs** **(*****n*** = **192)**		**NICUs** **(*****n*** = **122)**		**Physicians** **(*****n*** = **100)**		**Nurses** **(*****n*** = **145)**		**Others** **(*****n*** = **69)**	
	***N***	**%**	***N***	**%**	***N***	**%**	***N***	**%**	***N***	**%**	***N***	**%**
Sex												
Females	281	89.5	182	94.8	99	81.1	73	73.0	140	96.6	68	98.6
Males	33	10.5	10	5.2	22	18.0	27	27.0	5	3.4	1	1.4
	**Mean**	**SD**	**Mean**	**SD**	**Mean**	**SD**	**Mean**	**SD**	**Mean**	**SD**	**Mean**	**SD**
Job experience (years)	18.74	10.61	19.34	10.55	17.78	10.73	17.13	10.57	22.37	9.46	13.42	10.27

**Table 2:** Bivariate correlations among study variables and emotional distress index.

	**Emotional distress index**		
	**Whole sample**	**NWs setting**	**NICUs setting**
COPE problem solving	0.009	−0.006	0.030
COPE positive attitude	−0.074	−0.016	−0.166
COPE trascendent orientation	0.097	0.172^*^	0.006
COPE social support	0.142^*^	0.111	0.183^*^
COPE avoidance strategies	0.362^**^	0.384^**^	0.332^**^
SAQ team climate	−0.076	−0.034	–**0.160**
SAQ safety climate	−0.099	−0.075	−0.153
SAQ job satisfaction	–0.112^*^	−0.066	–0.222^*^
SAQ stress recognition	0.281^**^	0.300^**^	0.256^**^
BIS	0.304^**^	0.311^**^	0.292^**^
BAS-R	0.034	0.055	−0.007
BAS-F	0.164^**^	0.053	0.295^**^
BAS-D	0.123^*^	0.066	0.189^*^

Note: Bold values indicate significant (*p* < 0.05) results.

* *p* < 0.05;

** *p* < 0.01.

**Table 3:** Descriptive statistics (raw values) and *t*-test results comparing professionals from neonatal wards (NWs) and neonatal intensive care units (NICUs) on study variables.

	**Total** **Mean (SD)**	**NWs** **Mean (SD)**	**NICUs** **Mean (SD)**	***t*** **-Test (*p*)**
COPE problem solving	2.97 (0.53)	2.97 (0.55)	2.98 (0.48)	−0.32 (0.746)
COPE positive attitude	3.03 (0.52)	3.02 (0.53)	3.04 (0.49)	−0.30 (0.761)
COPE transcendent orientation	1.94 (0.97)	1.97 (0.97)	1.89 (0.99)	−0.87 (0.383)
COPE social support	2.70 (0.68)	2.68 (0.69)	2.73 (0.65)	−0.77 (0.442)
COPE avoidance strategies	1.48 (0.46)	1.48 (0.46)	1.48 (0.45)	−0.32 (0.750)
SAQ team climate	3.21 (0.82)	3.19 (0.88)	3.23 (0.72)	−0.72 (0.490)
SAQ safety climate	3.34 (0.75)	3.32 (0.79)	3.37 (0.68)	−0.98 (0.352)
SAQ job satisfaction	3.72 (0.82)	3.66 (0.88)	3.83 (0.71)	−2.04 (0.043)
SAQ stress recognition	3.32 (1.02)	3.31 (1.04)	3.33 (0.99)	−0.18 (0.857)
BIS	3.34 (0.68)	3.31 (0.68)	3.37 (0.69)	−0.62 (0.536)
BAS-R	3.78 (0.81)	3.73 (0.79)	3.87 (0.82)	−1.41 (0.161)
BAS-F	2.80 (0.85)	2.73 (0.83)	2.92 (0.88)	−1.84 (0.066)
BAS-D	2.25 (0.88)	2.22 (0.85)	2.30 (0.94)	−0.61 (0.545)

### Regression analyses

Results of the linear regression analyses used to test the association between personality, coping and safety culture with the EDI are summarized in Table 4. The final model accounted for a significant proportion of the variance in the EDI [*R*^2^ =0.25; F (5,308) = 21.865; *p* < 0.001]. Higher avoidance strategies, BIS sensitivity, BAS-F and SAQ stress recognition were all associated with higher EDI (Beta's range = 0.14–0.30, *p* < 0.005). SAQ job satisfaction was negatively associated with the EDI (Beta = −0.12, *p* = 0.017). All other predictors were excluded from the equation as not statistically significant.

**Table 4:** Multiple linear regression model predicting the emotional distress index.

	***ΔR^2^***	**B**	**SE**	**Beta**	***t***	***p***
COPE avoidance strategies	0.13	1.73	0.29	0.30	5.97	<0.001
SAQ stress recognition	0.07	0.78	0.20	0.20	3.86	<0.001
BIS	0.03	1.24	0.33	0.20	3.73	<0.001
BAS-F	0.01	0.59	0.21	0.14	2.82	0.005
SAQ job satisfaction	0.01	−0.62	0.26	−0.12	−2.40	0.017

B, unstandardized coefficients; Beta, standardized coefficients.

Importantly, the association between personality, coping and safety culture and EDI differed depending on working setting. In NWs a model that includes COPE avoid, BIS and SAQ stress recognition explain a significant proportion of the variance in EDI [*R*^2^ = 0.26; *F* (3,188) = 22.96; *p* < 0.001], with higher scores on these scales being associated with greater emotional distress in health care professionals. Differently, in NICU professionals a significant proportion of the variance in EDI [*R*^2^ = 0.27; *F* (4,117) = 12.34; *p* < 0.001] was predicted by a four-predictor model that include COPE avoid, BIS, BAS-F and SAQ job satisfaction. COPE avoid, BIS, BAS-F were all positively related to the EDI (Beta's range = 0.23, 0.30, *p* < 0.005), whereas SAQ job satisfaction was negatively associated with this score (Beta = −0.24, *p* = 0.003).

### Cluster analysis

**Figure 1:**
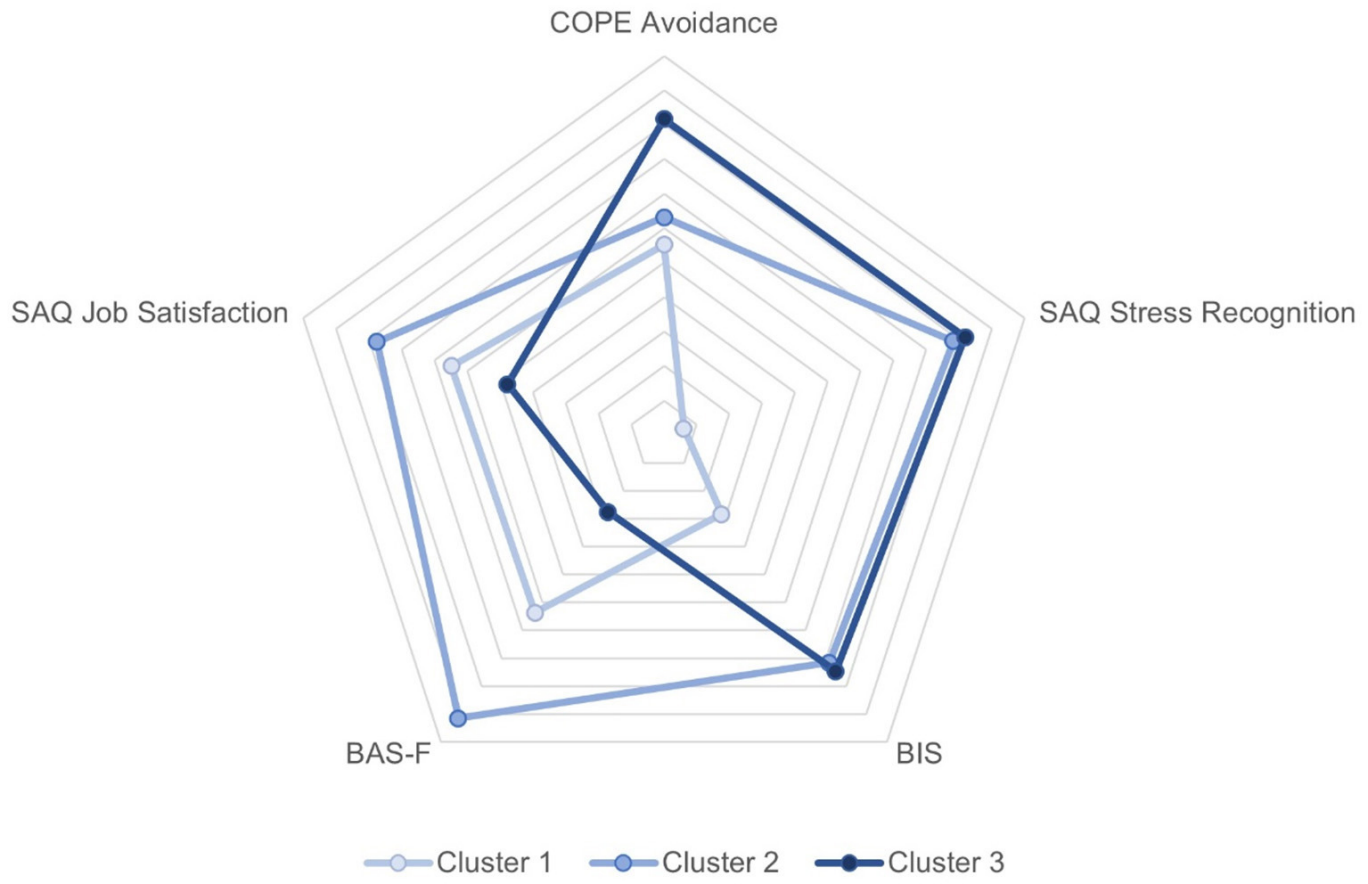
Radar plot of mean scores on personality, coping and safety culture by clusters. A greater distance from the center represents a more positive score on the scale.

A two-step cluster analysis was performed including all the significant predictors of EDI emerged from the regression analysis in the whole sample. A three-group solution was found with the following distribution: 23.6% (*n* = 74) of the participants in Cluster 1, 45.9% (*n* = 144) in Cluster 2 and 30.6% (*n* = 96) in Cluster 3. As summarized in Table 5 and shown in Figure 1, the first group resulting from the cluster analysis (Cluster 1) was characterized by low scores on BIS, COPE avoidance strategies and SAQ-Stress recognition, moderate levels of BAS-F and SAQ job satisfaction. The second group (Cluster 2) identified professionals with high levels of BAS-F, BIS, SAQ stress recognition and SAQ Job satisfaction while low COPE avoidance strategies. The third group (Cluster 3) clusters professionals with high scores in COPE avoidance strategies, SAQ Stress Recognition and BIS, while low scores on BAS-F and SAQ job satisfaction.

**Table 5:** Descriptive statistics for professionals from the three clusters on study variables.

	**Cluster 1** **Mean (SD)**	**Cluster 2** **Mean (SD)**	**Cluster 3** **Mean (SD)**
COPE avoidance strategies	1.35 (0.36)	1.42 (0.37)	1.68 (0.57)
SAQ job satisfaction	3.64 (0.90)	4.01 (0.55)	3.36 (0.94)
SAQ stress recognition	2.02 (0.61)	3.69 (0.74)	3.77 (0.76)
BIS	2.77 (0.49)	3.50 (0.67)	3.54 (0.61)
BAS-F	2.69 (0.74)	3.34 (0.60)	2.08 (0.68)

**Figure 2:**
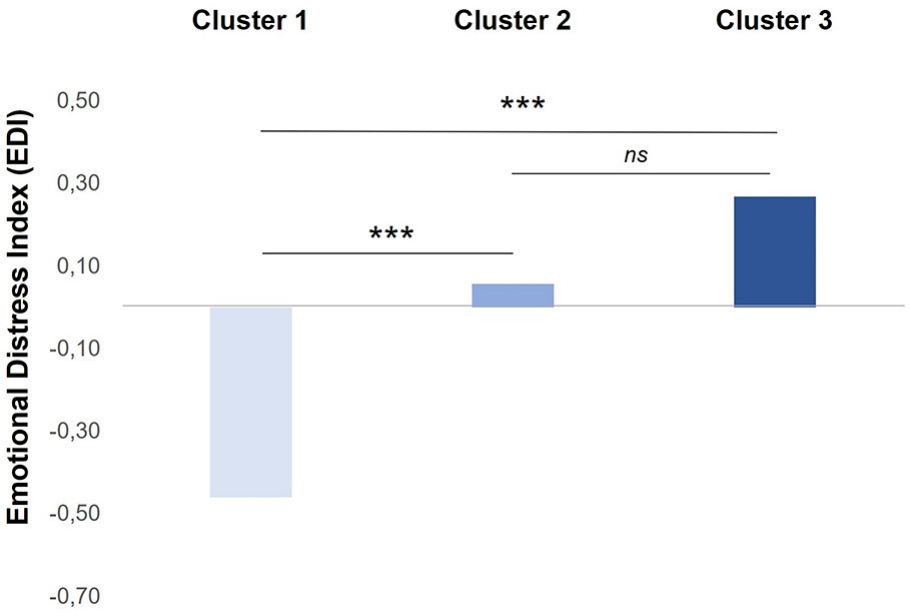
Mean scores on EDI by clusters. ***, *p* < 0.001. EDI, emotional distress global index.

After classification into groups based on the three-cluster solution, a univariate analysis of variance (ANOVA) was performed to investigate differences in the EDI scores between clusters. The results of the comparative analysis between clusters on the EDI (Figure 2) demonstrated statistically significant differences among clusters [*F* (2,311) = 12.36, *p* < 0.001]. *Post-hoc* Scheffè-adjusted comparisons showed that Cluster 1 had a significantly lower score on the EDI (M = −0.46, SD = 0.76) than Cluster 2 (M = 0.06, SD = 0.99) and Clusters 3 (M = 0.27, SD = 1.06).

## Discussion

This study examined the relative contributions of personality, coping factors and safety culture on healthcare professionals' emotional distress in neonatal low-risk (i.e., NWs) and high-risk (i.e., NICUs) settings. BIS/BAS sensitivity, avoidance coping strategies, stress recognition and job satisfaction were all significant predictors of workers' mental health outcomes, including depression, anxiety, psychosomatic symptoms, post-traumatic stress symptoms and emotional exhaustion. Further, we showed that these associations varied according to the working settings (i.e., NICUs vs. NWs). Lastly, an exploratory cluster analyses revealed three specific profiles of professionals in term of personality, coping and safety culture, which further predicted individuals' emotional distress.

In line with our prediction, greater BIS sensitivity, BAS fun-seeking sensitivity, avoidance coping strategies and stress recognition were all associated with greater emotional distress in the whole sample. BIS and BAS dimensions have been previously associated with emotion regulation difficulties (20) and implicated in the development of psychopathology in the general population (46). The current study extends this evidence by showing, for the first time, that both high BIS and BAS fun-seeking are associated with emotional distress in healthcare professionals. Strong empirical support exists for an association between high BIS sensitivity and anxiety (21), depression (47), and post-traumatic stress symptoms (48). In contrast, the BAS dimension is less well-understood (49). High BAS sensitivity has been consistently associated with the externalizing dimension of psychopathology (50), whereas evidence concerning the link with internalizing symptoms is mixed (21, 51–53). The multi-faceted nature of the BAS construct may partly explain inconsistencies in the literature (49). While BAS Reward Responsiveness subscale has been found to uniquely predict lower behavioral problems and heightened psychological wellbeing (54), the BAS-Fun seeking subscale is thought to reflect a more maladaptive dimension of BAS encompassing impulsivity and lack of control (54, 55). The current findings support this hypothesis by showing that high BAS-Fun seeking predict greater emotional distress in healthcare professionals. Importantly, BAS-Fun Seeking, but not the other BAS subscales, has been positively associated with self-reported emotion regulation difficulties (56), dysfunctional impulsivity (49), experiential avoidance strategies (57) and substance use (58). We might hypothesize that seeking rewards or pleasurable experiences may lead, in the long term, to maladaptive consequences for individuals' mental health. However, this remains an open question for future studies adopting a longitudinal design.

As anticipated, avoidant coping strategies were related to greater emotional distress among healthcare professionals. This result is in line with previous literature on the link between avoidant coping and emotional distress (27, 59). Coping is a critical aspect of person-environment transactions that occurs when an individual appraises a situation as stressful (60). The current finding suggests that adopting strategies based on avoidance when faced with stressful situations represents a dysfunctional response and might increase the risk of psychological problems in neonatal healthcare professionals. As these working contexts, particularly within the NICUs, are inherently characterized by high levels of stress, improving professionals coping strategies should be a priority.

Safety attitudes were significantly associated with professionals' mental health problems in the current study. Specifically, greater stress recognition (i.e., acknowledgment of how performance is influenced by stressors) was related to an increase in emotional distress, whereas job satisfaction predicted lower distress. This is in line with a previous work showing an association between stress recognition and anxiety, depression, and burnout in healthcare professionals during the pandemic (35). Likewise, evidence suggests that job satisfaction plays a protective role against emotional disturbances in healthcare professionals (8, 35, 61). Although the direction of these associations is unclear, they highlighted that safety attitudes are important indicators of professional wellbeing. Importantly, safety attitudes are modifiable and sensitive to quality improvement interventions (62). Institutional initiatives that sustain safety culture and particularly job satisfaction may promote professionals' psychological wellbeing and thus improve caring processes.

Critically, the current study suggests differential associations between personality, coping and safety culture and professionals' emotional distress in low-risk (i.e., NWs) and high-risk (i.e., NICUs) working contexts. While avoidant coping strategies and BIS sensitivity were risk factors for professionals' psychological wellbeing in both settings, SAQ stress recognition predicted greater emotional distress in NWs only, whereas BAS sensitivity and job satisfaction were significant contributors of professional's mental outcomes in NICUs. It is important to emphasize that the current findings are exploratory and are based on a sample of healthcare workers with mild-to-moderate levels of emotional distress. Replication in larger and clinical samples of healthcare workers with full-blown emotional disorders is needed. It has been well-documented that hospital intensive care units are inherently stressful work environments (63). It can be hypothesized that the stress-related nature of the working context might interact with individuals' characteristics to determine mental health outcomes (64, 65). The current findings are in line with previous evidence which showed a protective role of job satisfaction, while no effects of stress recognition, on emotional distress in professionals working within NICUs (8, 37). Critically, the degree of emotional distress of care providers has been related to healthcare-associated infection rates (9, 66) and adverse events within the NICUs (67), with the neonates admitted to NICUs being more vulnerable to the negative impacts of medical errors and adverse events compared to other patients (68). Thus, enhancing professionals' job satisfaction might be an important intervention target, particularly within the NICUs, in order to mitigate the consequences and the likelihood of these events.

Furthermore, findings showed that high BIS and high BAS-Fun seeking are associated with an increased risk of emotional disturbances for workers within the NICUs, but not within NWs. NICUs providers are exposed to extreme life experiences and daily controversial ethical decision. We might speculate that when facing this kind of stressful experiences individuals that are high on both BIS and BAS may frequently find themselves in approach–avoidance conflicts due to the potential threats and rewards present which may, in turn, lead to high levels of distress. More robust research is needed to understand the mechanisms underlining the observed association as well as the interplay between stress at the workplace and individual personality traits to determine health outcomes.

Lastly, in an exploratory cluster analysis we identified three different profiles of professionals based on personality, coping and safety attitudes. The first one had low scores on all the dimensions analyzed, except for job satisfaction. The second profile referred to professionals that show few avoidant coping strategies but were high on all other dimensions. Finally, the third group consisted of providers with high levels of avoidance, BIS and stress recognition, while low levels of job satisfaction and BAS. Importantly, professionals from the first group reported lower levels of emotional distress than those from the other two groups. Albeit preliminary, these findings suggest that a combination of low levels of BIS/BAS, low avoidance, low stress recognition and high job satisfaction significantly contribute to professional's wellbeing. A psychologically healthy workforce may promote patient safety and quality of care.

Some limitations of the present study are noteworthy. First, results are based on a sample of self-selected professionals, which may bias our findings in an unpredictable direction. Second, we included only a number of NW and NICUs from Tuscany, thus limiting generalizability to different units or locations. Third, we relied on self-report for all measures, which may have artificially inflated observed associations due to shared method variance. Fourth, while the influence of a number of potential confounders (i.e., sex, job, years of experience) on the observed associations was statistically tested, we cannot rule out the effects of other potential psychological and working determinants of emotional distress (such as previous mental health problems, lifestyle variables, hours of overtime work, and/or work overload) which remains open questions for future investigations. Lastly, although it is tempting to interpret the observed associations as suggestive of causative pathways, the cross-sectional and observational design does not allow to establish any causal relationship (69).

## Conclusion

Studies conducted in neonatal and pediatric care units have commonly reported high levels of mental health problems among the professional team (8, 15, 16, 70). This study identified a number of factors related to the personality, coping strategies and safety attitudes that contribute to the psychological wellbeing of professionals working in these contexts. The precise mechanisms of the observed associations require further exploration through prospective studies. However, the protective role of job satisfaction suggests that this might be an important intervention target. For example, the literature suggests that job satisfaction can be enhanced by implementing effective performance assessment processes, facilitating communications between professionals and patients, particularly in the case of adverse events, and improving professionals' sense of belonging and involvement (71, 72). In a clinical area where healthcare workers are satisfied with their job, they are likely to be less emotionally distressed and, thus, more likely to deliver safe and high-quality care and be mindful of their patients' needs.

## Data availability statement

The raw data of this article can be retrieved in Zenodo using this link: 10.5281/zenodo.7079093.

## Ethics statement

The studies involving human participants were reviewed and approved by Comitato Etico Regionale Sperimentazione Clinica della Regione Toscana. The patients/participants provided their written informed consent to participate in this study.

## The members of the SPACE-NET group

Roberta Cacciavellani, Giulia Placidi, Marzia Gentile, Armando Cuttano, Angelina Vaccaro, Claudia Maggi, Beatrice Gambi, Letizia Magi, Laura Crespin, Graziano Memmini, Cinzia Ceccarelli, Marcello DeFilippo, Elena Verucci, Roberto Danieli, Liliana Malandra, Giovanni Gaeta, Laura Mele, Pierluigi Vasarri, Elena Sandini, Angelo Azzarà, Patrizio Fiorini, Carlo Dani, and Alessandra Cecchi.

## Author contributions

LG and LP conceived the study. SG, SN, and LP made substantial contributions to the design of the work and drafted the manuscript. SC and IM organized and coordinated the acquisition of data for the work. SN performed the analysis and interpretation of the data and also prepared the first draft of the manuscript. All authors revised the manuscript critically for important intellectual content and approved the files for final submission.

## Funding

This study was funded by Regione Toscana (Bando Ricerca Regione Toscana COVID 19, Decreto, Dirigenziale Regionale n. 7731 del 26 maggio 2020).

## Conflict of interest

The authors declare that the research was conducted in the absence of any commercial or financial relationships that could be construed as a potential conflict of interest.

## Publisher's note

All claims expressed in this article are solely those of the authors and do not necessarily represent those of their affiliated organizations, or those of the publisher, the editors and the reviewers. Any product that may be evaluated in this article, or claim that may be made by its manufacturer, is not guaranteed or endorsed by the publisher.
